# Power Efficiency of Outer Hair Cell Somatic Electromotility

**DOI:** 10.1371/journal.pcbi.1000444

**Published:** 2009-07-24

**Authors:** Richard D. Rabbitt, Sarah Clifford, Kathryn D. Breneman, Brenda Farrell, William E. Brownell

**Affiliations:** 1 Department of Bioengineering, University of Utah, Salt Lake City, Utah, United States of America; 2 Marine Biological Laboratory, Woods Hole, Massachusetts, United States of America; 3 Department of Otolaryngology, Head and Neck Surgery, Baylor College of Medicine, Houston, Texas, United States of America; University of California San Diego, United States of America

## Abstract

Cochlear outer hair cells (OHCs) are fast biological motors that serve to enhance the vibration of the organ of Corti and increase the sensitivity of the inner ear to sound. Exactly how OHCs produce useful mechanical power at auditory frequencies, given their intrinsic biophysical properties, has been a subject of considerable debate. To address this we formulated a mathematical model of the OHC based on first principles and analyzed the power conversion efficiency in the frequency domain. The model includes a mixture-composite constitutive model of the active lateral wall and spatially distributed electro-mechanical fields. The analysis predicts that: 1) the peak power efficiency is likely to be tuned to a specific frequency, dependent upon OHC length, and this tuning may contribute to the place principle and frequency selectivity in the cochlea; 2) the OHC power output can be detuned and attenuated by increasing the basal conductance of the cell, a parameter likely controlled by the brain *via* the efferent system; and 3) power output efficiency is limited by mechanical properties of the load, thus suggesting that impedance of the organ of Corti may be matched regionally to the OHC. The high power efficiency, tuning, and efferent control of outer hair cells are the direct result of biophysical properties of the cells, thus providing the physical basis for the remarkable sensitivity and selectivity of hearing.

## Author Summary

The sense of hearing is exquisitely sensitive to quiet sounds due to active mechanical amplification of sound-induced vibrations by hair cells within the inner ear. In mammals, the amplification is due to the motor action of “outer hair cells” that feed mechanical power into the cochlea. How outer hair cells are able to amplify vibrations at auditory frequencies has been somewhat of a paradox given their relatively large size and leaky electrical properties. In the present work, we examined the power conversion efficiency of outer hair cells based on first principles of physics. Results show that the motor is highly efficient over a broad range of auditory frequencies. Results also show that the motor is likely controlled by the brain in a way that allows the listener to focus attention on specific frequencies, thus improving the ability to distinguish sounds of interest in a noisy environment.

## Introduction

Outer hair cells (OHC) in the mammalian cochlea are essential to the remarkable sensitivity of hearing. These highly specialized cells actively feed mechanical power into the organ of Corti and amplify its mechanical vibrations in response to sound [1]–[5]. How this is achieved at auditory frequencies is a subject of considerable debate. Five biological motor mechanisms have been described in outer hair cells that may contribute [2],[3],[5],[6]. Motors localized to the hair bundles include: actin-myosin motors associated with slow bundle movements and adaptation mechano-electrical transduction (MET) currents [7],[8]; Ca^2+^ sensitive reclosure or conformational change of the MET molecular apparatus associated with fast bundle movements and adaptation [9]; and electrically-driven bundle displacement that act independent of MET function [10]. Motors localized to the soma include: cytoskeletal remodeling mechanisms [11],[12] and electrically-driven changes in length [13]–[15]. The ability of each of these mechanisms to feed mechanical power into cochlea is limited by their intrinsic thermodynamic properties. As such, some of these motors can be ruled out as key to amplification of mechanical motions in the cochlea simply because they are too slow. The mammalian cochlear amplifier is extremely fast and capable of cycle-by-cycle action, in some species at frequencies exceeding 50 kHz [16],[17]. This rules out mechanisms that require cyclic phosphorylation, transport and/or protein synthesis. In non-mammalian species, that do not have OHCs or the protein prestin, bundle-based motors underlie the active amplification process [18],[19]. In mammals, the evidence indicates OHC somatic motility is a key contributor [20]–[24], and this is the motor we focus on here.

**Figure 1:**
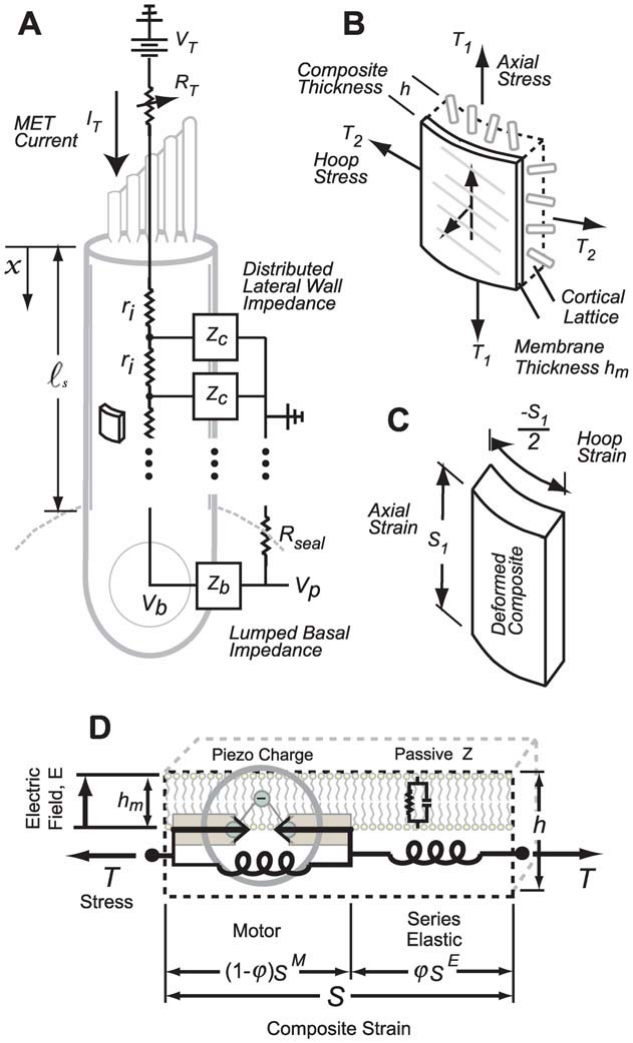
Model. A) The intracellular space was modeled as an axial conductor with resistance *r_i_* per unit length, intracellular voltage, *v(x,t)*, and axial displacement, *u(x,t)*. B) Axial and circumferential forces were assumed to be distributed across the cortical lattice/membrane complex of reference thickness, *h*, and represented by hoop, *T_2_*, and axial, *T_1_*, stresses. C) Isochoric deformations were assumed, thus relating the axial and circumferential strains, *S_1_* = −*S_2_/2*. D) The motor region of the lateral wall (*Z_c_* in panel A) was modeled as composite material consisting of passive elastic and active piezoelectric materials configured in series, with strains summating according to the mixture fraction 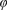 to give the composite strain. The base of the cell was modeled as a simple membrane with conductance and capacitance per unit area (*Z_b_* in panel A). Four stimulus conditions were simulated: sinusoidal transduction current injection *I_T_* entering the apical pole of the cell, sinusoidal displacement of the hair bundle leading to frequency-dependent MET currents, sinusoidal voltage clamp of the intracellular voltage *V_b_* at the base using a patch pipette and, sinusoidal modulation of the extracellular voltage *V_p_* at the base using a glass microchamber sealed with resistance *R_seal_* around the passive basal pole of the cell. In all simulations, cells were held stationary at 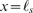 and generated force or movement at 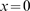.

OHC somatic electromotility is driven by the MET current entering the cell and likely draws thermodynamic power from the electo-chemical potential between fluid compartments in the cochlea. The apical surfaces of OHCs are bathed in high-potassium endolymph, biased to approximately +50 to +80 mV, and their basal poles bathed in high-sodium perilymph at 0 mV reference. This endocochlear potential is maintained by the stria vacularis and associated cells [25]–[27]. When the hair bundle is displaced and MET channels open at the tips the stereocilia, ionic currents (primarily K^+^ and Ca^2+^) are driven into the OHC. A fraction of this MET current enters the apical face of the soma at the base of the stereocilia. In the absence of phosphorylation, it is likely that this current carries the thermodynamic electrical power input that drives the OHC mechanical power output. Here, we analyze how this electrochemical energy is converted into useful mechanical work by somatic electromotility using the model illustrated in Fig. 1. The current model is fundamentally piezoelectric in nature and extends concepts developed by Iwasa [28],[29] to address frequency-dependent power conversion efficiency.

New results include the force vs. velocity, and power vs. velocity curves for OHCs (c.f. skeletal muscle cells [30]), and the frequency-dependent power efficiency that arises from intrinsic limitations on impedance matching between the cell and the load. Results indicate that OHCs are broadly tuned to have maximum power efficiency at a best frequency, thus contributing to tuning and the place principle in the cochlea. Furthermore, results provide an interpretation of how efferent activation may directly attenuate and de-tune the power output of OHCs and thereby providing a means for the brain to command exquisite control over the cochlear amplifier in a frequency dependent manner.

## Methods

Experimental procedures and animal care were designed to advance animal welfare and were approved by the Baylor College of Medicine animal care and use committee.

Our primary objective was to estimate what fraction of the electrical power entering the soma is converted into useful mechanical power output, and to estimate how this conversion efficiency would vary with frequency and biophysical parameters. It has not yet been technically possible to directly measure the electrical to mechanical power conversion efficiency of the OHC. The primary challenge is that one must measure the MET current, membrane potential, mechanical force generated and mechanical strain and velocity, all simultaneously and under physiologically relevant mechanical loading conditions. Therefore, we applied first principles of physics to formulate a relatively simple mathematical model of the OHC that reproduces all key published experimental data using a single set of physical parameters. The same model was then applied to compute the power conversion efficiency.

### 1. Model Derivation

#### Constitutive model for the lateral wall

The OHC lateral wall, where the motor elements are located [24],[31], was modeled as a series mixture of passive “elastic” and active piezoelectric “motor” elements [29] to arrive at a composite constitutive model for the lateral wall. We assume here that the passive portion of the lateral wall is associated with fraction, 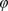, of the total lateral wall strain and the motor element is associated with fraction, 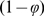. The fraction due to the motor was modeled as a leaky piezoelectric material. Following the notation of Tiersten [32], for the motor elements the stress tensor, 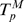, is related to the strain tensor, 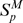, and the electric field, 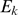, in the material according to
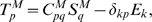
(1)where the superscript “*M*” denotes the motor element and the subscript “*p*” denotes the component of the stress tensor (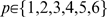). The tensor 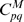 contains the elastic coefficients and the tensor 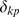 contains the piezoelectric coefficients. Einstein's summation convention applies for repeated indices. The electrical displacement current 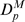 in the motor portion of the lateral wall is related to the strain and the electric field according to the constitutive model
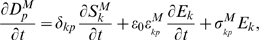
(2)where 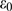 is the electrical permittivity of free space, 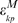 is the electrical relative permittivity, and 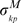 is the electrical conductivity of the motor portion of the lateral wall. The appearance of electrical conductivity is necessary to account for membrane electrical conductance makes Eq. 2 distinct from classical ideal piezoelectricity. The passive elastic component was modeled using the same approach, but with no piezoelectricity. In this case the stress tensor is
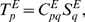
(3)and the displacement current is
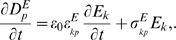
(4)The constitutive behavior of OHCs is nonlinear and included here by allowing the piezoelectric and elasticity tensors to depend upon the electric field and strain.

We further simplified the model by treating the OHC lateral wall as a thin shell undergoing axisymmetric deformations (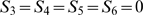; Fig. 1A–C), and assumed the axial strain is related to circumferential strain by a negative 2×1 ratio (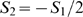; Fig. 1C). This strain ratio is consistent with experimental data [24] and, for small deformations, automatically enforces incompressibility of the intracellular volume (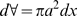; 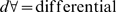 volume, *a* = cell radius, *dx* = differential length) for each differential slice of the OHC (i.e. 
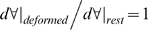
). To derive the series-composite model we assumed the axial stress to be identical in the motor and passive elastic components, 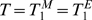, and that the total axial strain is found by series addition: 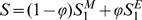. For the case when the elasticity tensors for the two materials are the same, the mixture parameter 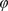 (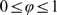) would be the fraction of the membrane surface area occupied by the passive elastic component, and the complement, 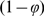, the fraction of the membrane area occupied by the motor. The electric field is dropped almost entirely across the plasma membrane and therefore varies through the thickness of the composite. After algebra, Eqs. 1–4 simplify to give the axial stress, *T*, in the composite
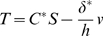
(5)where upper case 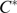 is the composite material stiffness, *S* is the overall axial strain, 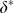 is the composite piezoelectric coefficient, *h* is the reference thickness of the composite lateral wall, and *v(x,t)* is the perturbation in membrane potential from the resting potential. The electrical current per unit membrane area, *i_m_*, is related to the electric charge displacement by 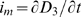 and given by:
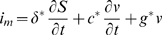
(6)where lower case 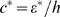 is the capacitance per unit area under zero strain that arises from the membrane permittivity, and 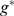 the membrane conductance per unit area. The effective permittivity of the composite, 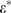, is referenced in the present analysis to the composite thickness, *h*. Since the passive capacitance arises almost exclusively from the plasma membrane, 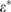 of the composite is related to the permittivity of the plasma membrane, 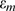 by 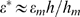 (under the special case 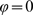 as discussed below). We note that the composite thickness, *h*, always appears as a product with physical parameters, and therefore can be selected as the membrane thickness or a larger value incorporating the cortical lattice with equivalent results (providing the related parameters are scaled appropriately as discussed below). Eq. 5–6 are shown in their linearized form where the stress *T*, strain *S*, electric field *E*, and the charge displacement *D* are small perturbations from the resting state (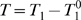, 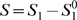, 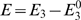 and 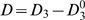, the superscript “0” refers to values in the resting state). In accordance, the physical parameters in Eq. 5–6 are the linearized values about the resting state (composite stiffness 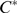, composite piezoelectric coefficient 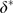, composite capacitance 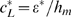, composite conductance 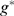, all evaluated at the resting state: 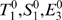 and 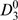). We also note that perturbations in the stain are related to axial displacement from rest *u(x,t)* by 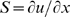, perturbations in stress are related to changes in axial force *f(x,t)* by 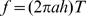, and perturbations in the electric field are related to changes in voltage *v(x,t)* by 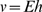. Accordingly, the axial force generated by the cell is related to the displacement and voltage by 
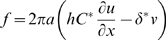
 and the axial current is related to the gradient of the voltage by 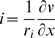.

This composite material is distinct from ideal piezoelectric materials due to the presence of a membrane conductance, and because the motor itself only occupies a fraction of the membrane surface area while the remainder is occupied by passive elastic material [32],[33]. It is important to note that the composite properties are related to the mixture fractions and properties of the motor and elastic constituents. The composite elasticity of the series-composite lateral wall in the axial direction is 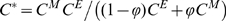, and the composite piezoelectric coefficient is 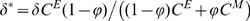. Maxwell piezoelectric reciprocity applies for each of the constituent materials and for the series-composite (Eq. 5–6) – consistent with OHC data [34],[35]. The capacitance per unit area of the series composite under conditions of zero strain is 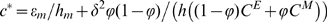, where 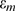 is the electrical permittivity of the plasma membrane. Note that the piezoelectric coefficient 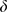 contributes to the passive capacitance even in the zero strain case. This is because series expansion of the motor element can be offset by contraction of the passive element thus resulting in zero composite strain but non-zero piezoelectric charge displacement. This is distinct from ideal piezoelectric materials, but necessary when modeling OHCs to account for voltage-dependent capacitance in model cell lines observed even under zero overall strain (e.g. prestin transfected HEK cells [36]). We also found that standard piezoelectric materials were unable to simultaneously match the capacitance, displacement, and force observed in OHC, while the composite model was capable of matching all of the data with a single model parameter set. Since the piezoelectric coefficient 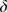 is voltage-dependent, the area-specific capacitance 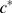 is also voltage-dependent and cells exhibit nonlinear capacitance even when the strain is zero. Present simulations assume the membrane permittivity, 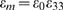, is not voltage dependent and is spatially uniform. Although it is not difficult to include, present results therefore do not address voltage dependence of the linear capacitance or any influence prestin configuration might have on this [36].

In piezoelectric materials occurring in nature, the coefficient 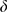 is a function of strain and saturates for large strains. This occurs because of kinematic constraints on the molecular configuration within the material that limits the strain range of the piezoelectric effect. The strain-dependent saturating effect in OHCs follows this rule in that OHCs simultaneously exhibit length changes and charge movements upon varying the holding potential [37] and/or intracellular turgor pressure [38]. Piezoelectric saturation is more easily observed in OHCs experimentally using command voltages [36],[39],[40] due to the difficulty of strain controlled experiments. Because of this, we modeled the piezoelectric coefficient as dependent upon the holding potential using a Boltzmann function of the form 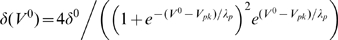, where 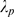 is the variance associated with thermal motion, 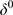 is the peak piezoelectric coefficient, 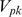 is the voltage at which the peak piezoelectric coefficient occurs and 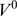 is the membrane potential about which the OHC model equations were linearized. This is the same form that has been routinely applied to describe OHC voltage-dependent capacitance (e.g. 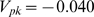 and 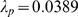) [36],[39]. We note that a voltage-dependence can be converted to a strain-dependence, under conditions of zero change in stress, using the piezoelectric constitutive Eqs 5–6. Hence, an intrinsic strain-dependent piezoelectric coefficient associated with a change in molecular configuration can be observed experimentally as voltage-dependence.

#### Conservation of momentum (Newton's 2^nd^ law)

Conservation of momentum in the axial direction can be written [41],[42]

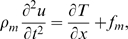
(7)where 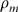 is the density of the composite membrane material and 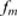 is the fluid drag shear stress per unit length acting on the membrane from the extra- and intra-cellular fluids. Substituting the axial stress from Eq. 5 and the fluid drag from Section 2 into Eq. 7 provides
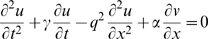
(8)where *u(x,t)* is the local axial displacement of the membrane, *v(x,t)* is the perturbation in the local membrane potential, *x* is the axial position along the cell, *t* is time, 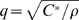 is the passive mechanical wave speed in a vacuum, 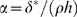 (∼−80 m^3^/volt-sec^2^) is a piezoelectric coefficient, and 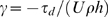 (see below) is a damping coefficient resulting from immersion of the cell in fluid. If the voltage is uniform in space or the piezoelectric coefficient is zero, Eq. 8 reduces to the classical mechanical wave equation [41]. For OHCs the mechanical equations are overdamped such that propagating waves decay and sharp mechanical resonance is not expected [43]. If energetically favorable frequencies do exist, they would be electromechanical in nature and not strictly mechanical [44]. Also, at auditory frequencies, the first term (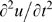) is small and could be ignored relative to other terms (retained in the present simulations).

#### Conservation of charge (Kirchhoff's current law)

Electrically, the OHC was modeled as a cylinder filled with conducting cytoplasm and immersed in a conducting fluid media (Fig. 1). The extracellular media was assumed to be space clamped and grounded (zero voltage), but the intracellular fluid voltage *v(x,t)* was allowed to vary with axial distance “*x*” from the apex and with time “*t*”. Current entering the MET channels was assumed to travel in the axial direction from the apex to the base. This configuration creates a current divider, with one fraction of the current directed out the base of the cell while the other fraction drives the lateral-wall motor. For simplicity, we consider the idealized case where the motor is modeled as homogeneously distributed along the lateral wall. We modeled the intracellular voltage using the same approach used for passive axons, reviewed by Weiss [45], but we replaced the classical plasma membrane electrical impedance with the series-composite model (Eq. 6) to obtain:
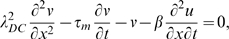
(9)where 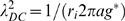 (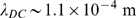; 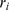 intracellular axial resistance per unit length in Ohm/m, 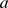 cell radius and 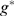 membrane conductance per unit area in S/m^2^) is the DC electrical space constant analogous to that in the standard cable equation, 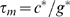 (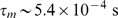) is the composite membrane time constant under zero deformation, and 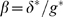 (∼0.075 volt-s; 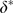 composite piezoelectric constant) is a piezoelectric coefficient coupling the piezoelectric charge movement to strain. Eq. 9 reduces to the standard cable equation used for passive axons in the absence of strain and/or piezoelectricity [45].

#### Experimental conditions simulated

Four types of stimuli and three loading conditions were considered. For stimuli, we considered voltage clamp (VC) of the basal region of the cell (*V_b_* specified in Fig. 1), current injection at the apex of the cell simulating a constant amplitude sinusoidal MET currents (*I_T_* specified in Fig. 1), micro-chamber (MC) control of the extracellular voltage surrounding the basal pole of the cell (*V_p_* specified in Fig. 1) [24],[46], and hair bundle displacement leading to an adapting MET current (*I_T_* from Eq. 10 below) [47]. For boundary conditions, we considered isometric loading (zero displacement at 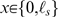), zero-load displacement (force zero at 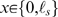), and the ideal intermediate case where the OHC was loaded in a way to achieve maximum mechanical power output.

#### MET current adaptation

In a subset of simulations we estimated the velocity and force for physiological hair bundle movements. The OHC transduction current appears to adapt very rapidly to step hair bundle displacements (time constant on the order of 100 micro-seconds), and the adaptation may be nearly 100% complete in some cells [47]. Although adaptive responses of OHC transduction currents are nonlinear, a simple first-order linear adaptation model captures some of the major features:
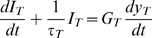
(10)where the transduction current is 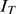 (Amp), the adaptation time constant is 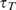, the transduction current gain is 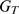; 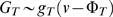, 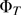 is the transduction current electrochemical potential, and the hair bundle displacement is 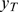 (m). The transduction current was set equal to axial current at the apical end of the cell and related to the voltage gradient along the cell at its apex using 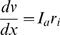 where 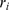 is the intracellular axial resistance. Adaptation causes the current to increase as the frequency is increased, at least for stimuli below 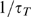 (rad/sec). This counters the capacitance of OHCs and thus would be expected to flatten the frequency response of the intracellular voltage relative to responses to sinusoidal current injection.

#### Analytical solution

Equations 8–10 define the model and were solved in the frequency domain using an eigenvector expansion. The model equations were solved by first considering the a solution in the form: 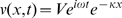 and 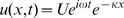, where 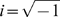, 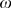 (rad/sec) is the stimulus frequency, and 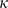 is an eigenvalue. Substitution into Eqs. 8–9 provides a 4^th^ order eigenproblem
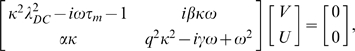
(11)which yields, at each frequency, four eigenvalues 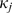 and corresponding eigenvectors 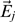. The frequency-dependent AC space constant under which each piezoelectric eigenwave propagates is 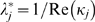, and the phase velocity is 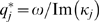. Having these eigenvalues, we write the general solution for a finite length OHC in the form of an eigenvector expansion
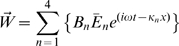
(12)where the frequency-domain voltage and displacement are components of 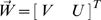. The four independent eigenvectors are 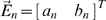, with corresponding eigenvalues 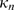.

The coefficients 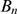 are found from four boundary conditions. To model the *isometric* condition, we require the displacement at the two ends of the OHC lateral wall to be zero (at 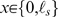). From Eqs. 5 and 11 this gives two equations
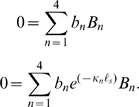
(13)For the *zero-force* condition, we require the stress to vanish at the ends of the lateral wall (at 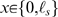) to find
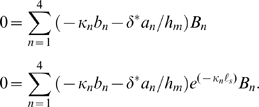
(14)To close the problem, we need two additional boundary conditions. In most simulations we drive the OHC *via* a sinusoidal current injection at the apical end of the cell, *x* = *0*. Under this condition the intracellular voltage gradient is related to the current injection 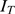 at the apex and the axial resistance per unit length according to 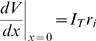. Substitution into Eq. 11 gives
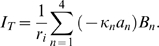
(15)At the other end of the cell, current exits the region adjacent to the lateral wall motor and enters the basal compartment – a compartment we model using a lumped impedance *Z_b_* at the base of the cell. For this case
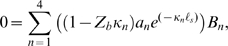
(16)For a voltage clamp simulations, we specify the intracellular voltage *V_b_* at the base of the cell
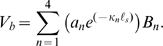
(17)Stimulation of the OHC by modulating the voltage *V_p_* in a pipette microchamber enveloping the base of the cell [48] requires us to account for current through the membrane and gives
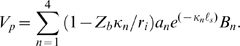
(18)After selecting isometric or zero-load conditions, and the stimulus type, the equations above provide 4 equations that are easily solved for the 4 unknown constants *B_n_*. Since the equations are linear, we use superposition to consider mixed boundary conditions as described below.

#### Power and Efficiency

The power efficiency was defined as the mechanical power output divided by the electrical power input. The mechanical power output is computed in the frequency domain using 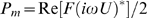, where 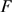 is force and 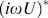 is the complex conjugate of the velocity, both evaluated at the apical end of the cell. The real part of the power output provides the time-averaged power transferred from the OHC to the external dissipative load. This real part of the power is the component that would be needed to overpower viscosity, for example. Similarly, the electrical power input *via* the MET channels is 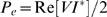, where *_V_* is the voltage drop across the MET and 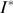 is the complex conjugate of the MET current. Since the model was linearized about the resting state, superposition of the isometric case and the zero-force case could be used to simulate any loading condition. By superposition, the force output by the cell is 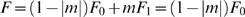, where *m* (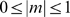) is a complex-valued parameter controlling the load, 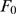 is the force under isometric conditions, and 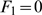 is the unloaded zero force condition (subscript 0 denotes the isometric case and 1 denotes the zero-force case). Similarly, the velocity is 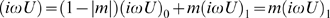. The corresponding MET voltage and current are 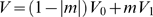 and 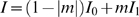, respectively. Combining these expressions gives the power conversion efficiency, 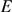, of the OHC as
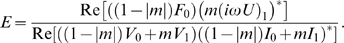
(19)Note that the efficiency is zero under isometric conditions (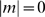) and is zero if no load is applied (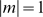). There is a unique load, magnitude and phase, that maximizes the efficiency. This “impedance-matched” load is frequency dependent and was found by solving for the complex-valued parameter *m* that maximized 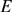.

The present model has some features similar to previous piezoelectric-like models of the OHC [29], [49]–[54], but the formulation differs by including a series elastic-piezoelectric composite constitutive model of the lateral wall and axial conductance of the intracellular space, and differs in considering power conversion from electrical power entering the transduction channels to mechanical power output to do useful work.

### 2. Visco-Elastic Fluid Drag

Dissipative drag from the cytoplasm and the extracellular space are unavoidable. As a first approximation we modeled the axial component of the drag acting on the plasma membrane using a version of the Navier-Stokes equations. Assuming small displacements from the resting configuration, and ignoring the convective nonlinearity, the Navier-Stokes equations reduce to
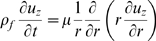
(20)where 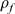 is the density of the fluid, *r* is radial coordinate, 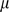 is the effective viscosity, and 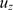 is the axial velocity. To approximate the visco-elastic properties of the materials, we used a complex-valued viscosity of the form 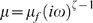, where 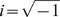, 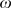 is the frequency, 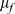 is a material constant, and the 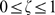 is a parameter that determines the relative contributions of viscosity vs. elasticity of the material. When 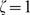 this model reduces to the standard Newtonian viscous fluid and when 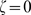 this reduces to the standard shear elastic solid. For biological materials *ζ* falls between these two extremes – e.g. 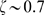 for the tectorial membrane [55]. These equations account for both the visco-elastic drag and entrained fluid mass. We solved the equations to obtain the velocity field 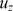 resulting when a cylinder oscillates in the axial direction with displacement 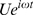. Having the velocity field, we computed the axial shear stress 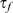 acting on the cylinder wall per unit axial displacement
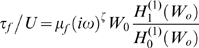
(21)where 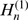 are Hankel functions, 
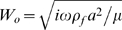
 is the non-dimensional Womersley number (complex-valued), and *a* is the cylinder radius. With this, the damping parameter appearing in the momentum equation (Eq. 8) is 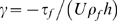. This model is approximate, but matches the viscous analysis of Tolomeo and Steel [43] if the length of the cell is much longer than the diameter, motions are axial, and the viscosity is strictly real valued, i.e. 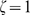.

### 3. Model Parameters

**Figure 2 pcbi-1000444-g002:**
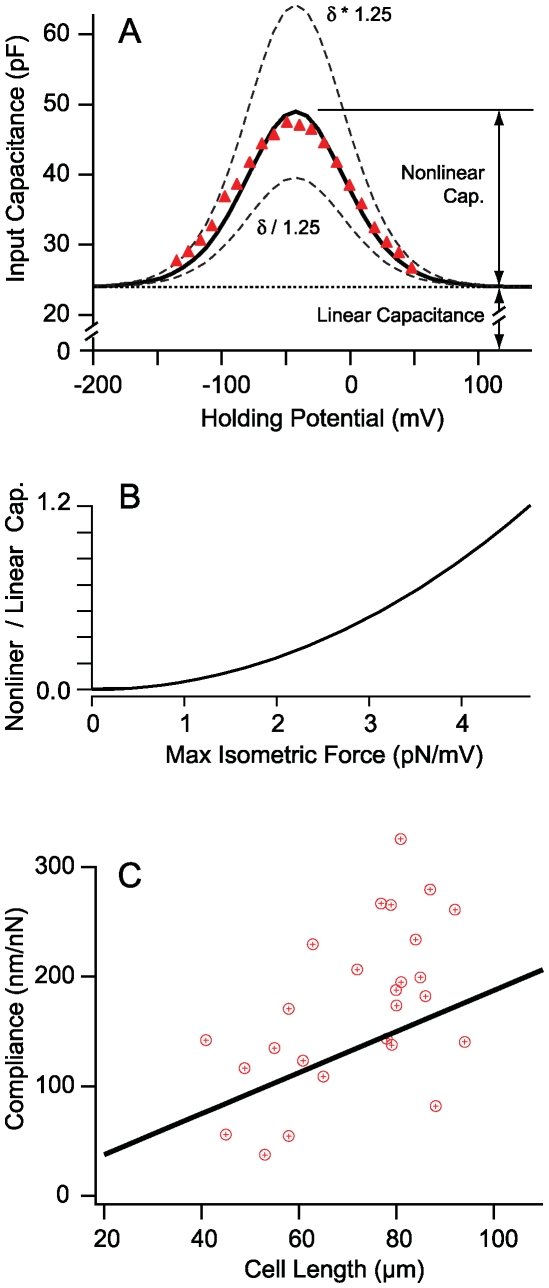
Voltage dependent capacitance and axial stiffness. A) Model predictions for the nonlinear capacitance based on the Boltzmann piezoelectric distribution compared to data from Kakehata & Santos-Sacchi [38] for a ∼50 micron long OHC under conditions of zero load. The capacitance exhibits a linear component plus a nonlinear (voltage dependent) component. Dashed curves show the effect of varying the piezoelectric coefficient by ±25%. B) The model predicts a parabolic relationship between the nonlinear component of capacitance and the peak isometric force as the membrane potential is traversed from −200 to +130 mV (same cell). All subsequent results are for small (linearized) forces and movements about a membrane potential of −78 mV. C) Compliance predicted by the model (solid line) is shown vs. cell length in comparison to data (symbols) from Frank et al., [48].

**Figure 3 pcbi-1000444-g003:**
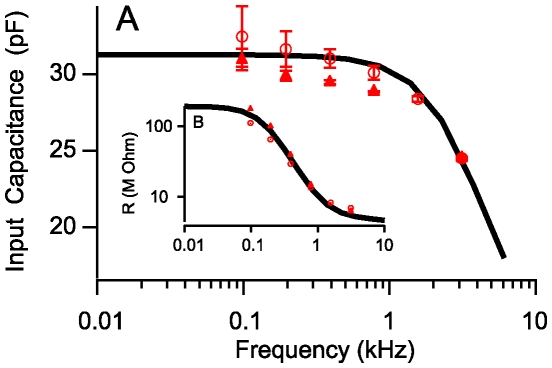
Input capacitance vs. frequency. A) Input capacitance and B) resistance of two 50 µm long OHCs measured with patch pipettes attached at the base begin to roll-off at high frequencies. Error bars denote one standard deviation of the capacitance at each frequency tested. Solid curves show model results. The capacitance begins to roll off above ∼1 kHz. The roll off is captured by the model due to a loss of space clamp that occurs at higher frequencies. These data were used to estimate the intracellular axial electrical resistance of the cell.

**Figure 4:**
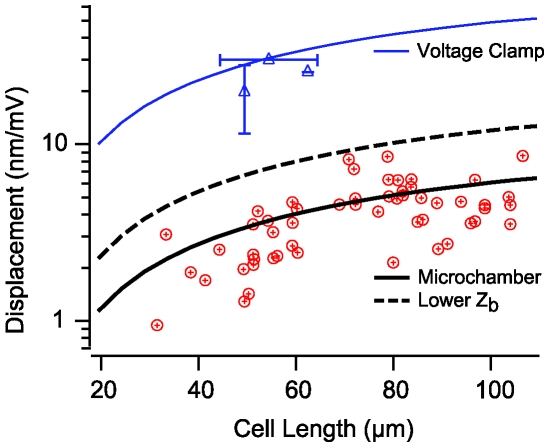
Displacement gain vs. cell length. Sinusoidal control of the extracellular voltage around the base of OHCs (microchamber configuration) evokes movment proportional to the voltage and dependent upon cell length. Symbols show microchamber data from Frank et al. [48] in comparison to the prediction of the present model (solid black curve, Eq. 18 at base). The same model simulated for voltage clamp conditions (solid blue curve, Eq. 17 at base) predicts voltage clamp data from Ashmore [15] and Santos-Sacchi, [62]. Also shown is the model prediction after increasing the basal membrane conductance by 2.2× (dashed curve, low Z_b_) to simulate application of Ach in the microchamber configuration. Hence, efferent action lowering *Z_b_* is predicted to increase OHC movement gain in the microchamber, but sharply attenuate the gain under physiological stimulation due to short circuit of the base of the cell.

**Figure 5:**
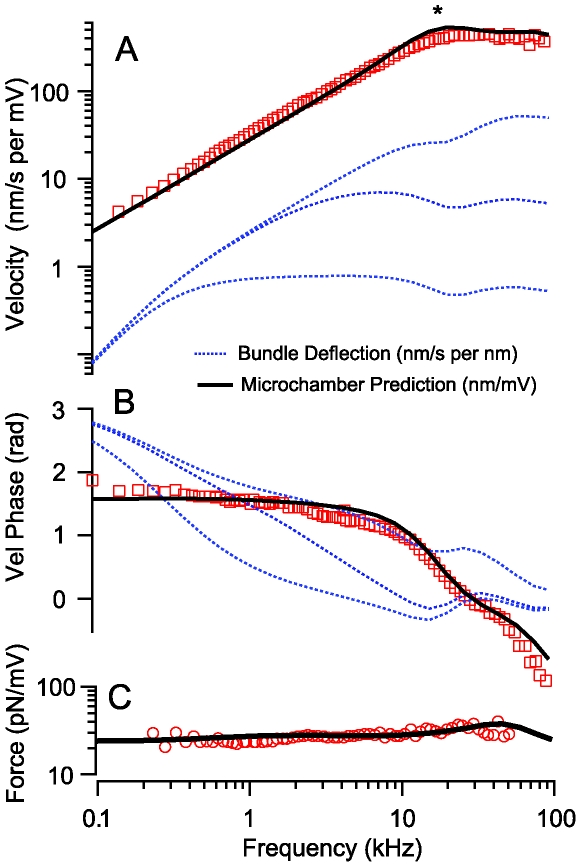
Axial velocity and isometric force vs. frequency. A) The zero-load velocity gain and B) phase are shown as functions of frequency for an 80 µm long OHC. Symbols replot data from by Frank et al. [48] (nm/s somatic velocity per mV extracellular microchamber voltage), and solid black curves provide the current model predictions, also in the microchamber configuration. The * denotes the OHC displacement corner frequency observed under microchamber conditions, which increases in value for shorter cells. Also shown are model projections for physiological hair bundle displacements (dotted, nm/s somatic velocity per nm of hair bundle displacement). The series of curves (blue dotted) show predictions for various rates of fast MET adaptation associated with the MET adaptation time constant (tT). Note that MET adaptation is predicted to introduce a broad-band phase roll-off and result in OHC velocity that increases with bundle displacement frequencies below 1/τ_T_ and becomes relatively flat for frequencies above 1/τ_T_. C) Isometric force generated by the same cell in the microchamber configuration (symbols) is predicted by the same model (solid black curve). Note the corner frequency is much higher under isometric force conditions due to the restriction on cell movement.

Model parameters were estimated from known dimensions and physical constants combined with voltage clamp and mechanical data shown in Figs. 2–3 as well as microchamber data in Fig. 4. All other results (Fig. 5–8) and voltage clamp data in Fig. 4 are model predictions and the associated data were not used to estimate parameters. The model uses a reference thickness 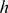 to describe the multi-component composite lateral wall and it is important to note that some parameters cannot be independently separated from this reference thickness (e.g. 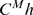, 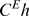, 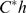 appear as groups). Coefficients appearing in the cable equation were computed from the physical parameters listed below using: 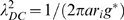, 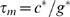, and 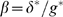. Coefficients appearing in the wave equation were computed using 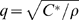, 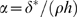 and 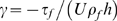. Dimensions were based on OHCs from the guinea pig cochlea. Data in Fig. 2–3 were used to find the effective stiffness, piezoelectric coefficient, electrical permittivity and conductance of the membrane. These data are for relatively low stimulus frequencies where the intracellular axial resistance has negligible effect on the results. To estimate the axial resistance we used the corner frequency where the capacitance measured at the basal pole of the cell begins to roll off (Fig. 3). The fraction of the membrane occupied by the motor was set to 80% (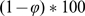) and the passive component to 20% (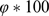). The overall cell compliance was estimated from the slope of the compliance vs. cell length reported by Frank et al. [48], reproduced in Fig. 2C, using 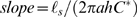 as well as the gain reproduced in Fig. 4 (solid, microchamber curve). An iterative optimization routine was run to refine the initial estimates of 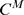, 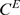 and 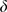 to simultaneously fit data in Fig. 2–4. Specific optimized numerical parameters include: OHC radius *a* = 4.5e-6 m; composite mechanical stiffness C* = 1.4e6 N/m^2^ (based on 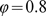, 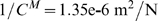 and 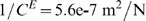); plasma membrane conductance 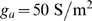; apical face membrane conductance 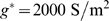; basal membrane conductance 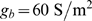; transduction current gain 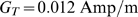; composite reference thickness 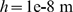; OHC length 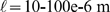; length of the active lateral wall was 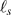, and 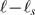 was set by requiring passive basal pole to have a passive capacitance of 7 pF; intracellular axial resistance *r_i_* = 5.76e10 Ohm/m; composite piezoelectric coefficient at rest 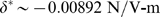 (C/m^2^) at rest; plasma membrane area specific capacitance 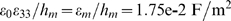; density 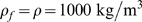; transduction current adaptation time constant 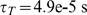; fluid viscosity 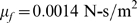; and fractional viscosity coefficient 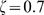. We note that the mixture fraction 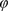 is not uniquely determined by currently available data and it is possible to find alternative mixture fractions and stiffness parameters that result in the same composite stiffness C*. Nevertheless, it was necessary to use a value of 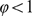 to simultaneously fit all of the data and explain the magnitude of voltage dependent capacitance under unloaded and zero strain conditions. Additional experiments, perhaps involving voltage-dependent capacitance measurements under controlled mechanical loads, have the potential to resolve this ambiguity and reveal more about the lateral wall motor, but are not necessary for the purpose of the present power analysis since the composite parameters would not change.

**Figure 6 pcbi-1000444-g006:**
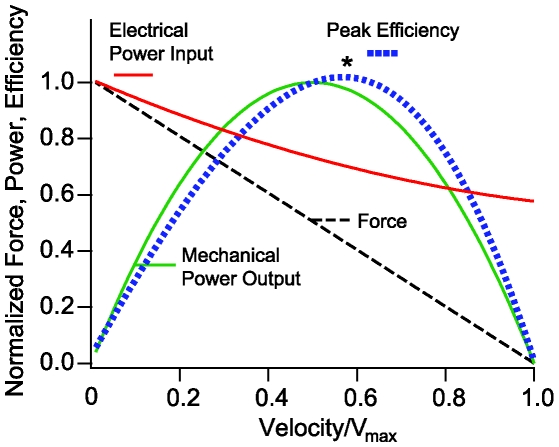
Normalized force, power, and efficiency vs. velocity. Maximum force is predicted to occur under isometric conditions (zero velocity), and maximum velocity is predicted to occur under zero load – both extremes require MET electrical power input but result in zero mechanical power output. The mechanical power output is shown as a function of velocity (solid parabolic curve) along with the electrical power input *via* the MET (solid red line). Efficiency is the ratio of the two curves (dotted curve) and peaks (*) at a force slightly lower that half of the isometric force and at the impedance-matched load corresponding to a velocity slightly higher than half of the zero-load velocity. This peak occurs at the “impedance matched” load.

**Figure 7 pcbi-1000444-g007:**
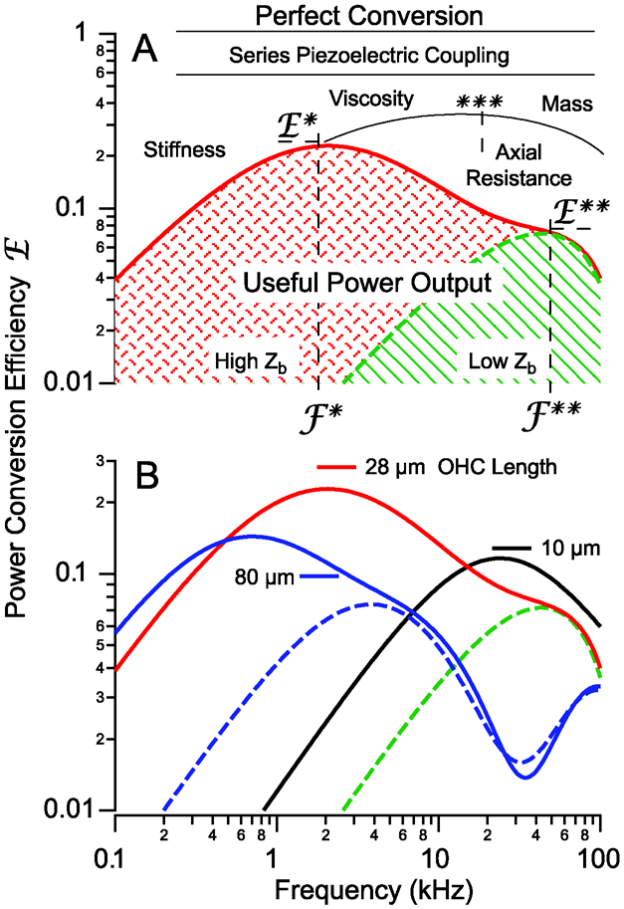
Power conversion efficiency. A) The taxonomy of electrical to mechanical power conversion efficiency delineating regions where input electrical power is lost to series-elastic piezoelectric coupling, OHC stiffness, fluid viscosity, entrained mass, and OHC intracellular axial electrical resistance for a 28 µm long cell. Results are shown under control conditions when the base of the OHC has a high impedance (solid red, cross-hatch, high *Z_b_*), and under conditions of low basal impedance associated with the action of efferent neurotransmitter on the base of the OHC (dashed green, diagonal hatch, low *Z_b_*). The peak efficiency 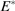 occurs at a best frequency 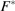, and shifts down in magnitude and up in frequency with opening of conductive ion channels in the basal cell membrane (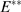, 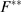). Hence, shunting of the basal impedance by efferent action on OHCs is predicted to attenuate their power output at best frequency 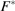, by well over an order of magnitude. B) The most efficient frequency depends upon cell length. Shorter cells show peak efficiencies at higher frequencies (10 µm) while longer cells show peak efficiencies at lower frequencies (80 µm). These predictions were computed by adjusting the load to be impedance matched at each frequency (peak efficiency load in Fig. 6 denoted by *).

**Figure 8:**
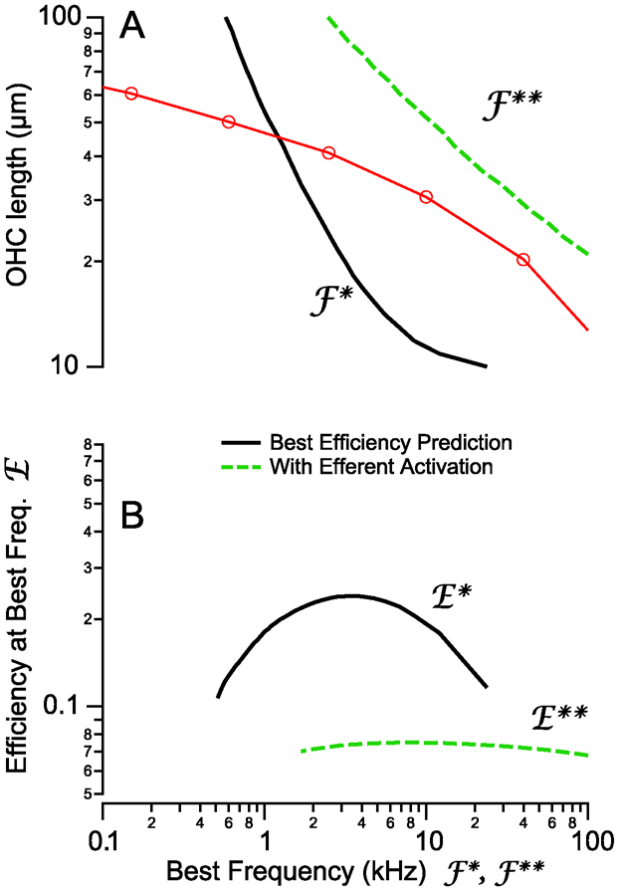
OHC length vs. best frequency. OHCs vary their length systematically with the place-principle of best frequency sensitivity in the cochlea. A) Anatomical lengths of hair cells in the cochlea (red symbols connected by lines, [78]) are compared to the length predicted by the present model to achieve maximum power conversion efficiency (frequency of peak in Fig. 7). Solid black curves show model predictions for peak efficiencies under control (high *Z_b_*) conditions while dashed green curves show predictions during efferent activation (low *Z_b_*). B) The model predicts that peak efficiencies vary systematically with OHC length, with cells tuned near 3–4 kHz being the most efficient (B, solid black curve). All cells are predicted to become inefficient when the basal electrical impedance (*Z_b_*) is reduced thorough activation of the efferent system (B, dashed green curve).

### 4. Experimental Methods

Experimental procedures and animal care were designed to advance animal welfare and were approved by the Baylor College of Medicine animal care and use committee. All physical parameters were deduced from the published literature, with the exception of the intracellular electrical resistance, 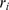. To estimate 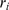, we isolated OHCs from the guinea pig cochlea [56] and examined the frequency dependence of the input electrical impedance under whole-cell voltage clamp (Axopatch 200 B, Molecular Devices, Sunnyvale, CA). OHCs were harvested from euthanized guinea pigs. Cells were patch-clamped at the base with quartz pipettes covered with Sylgard, and hyperpolarized to minimize the voltage-dependent nonlinear capacitance. K^+^ and Ca^2+^ ion channels were blocked with the addition of (C_2_H_5_)_4_N(Cl), CsCl and CoCl to the bathing and/or pipette solutions [57]. The input admittance was determined with a single sinusoidal voltage (0.015 V peak to peak, 90–3200 Hz) superimposed on top of a −0.13 V holding potential after correcting for the inherent phase shifts of the amplifier [58]. 210 measurements were averaged at each frequency. The resistance and capacitance were calculated from the input admittance [57] accounting for the series resistance (∼6 Mohm, remained constant throughout experiment). Experiments were conducted at room temperature.

## Results

### 1. Theory vs. Experiment

#### Voltage-dependent capacitance

When the lateral wall deforms there is a compensatory electrical charge movement due to deformation in the motor portion of the membrane – behavior that is fundamentally piezoelectric in nature [35]. Fig. 2A compares the voltage dependent input capacitance measured at the basal pole to model predictions (solid curves) for a 50 µm long cell tested using a small ∼100 Hz interrogation signal. Changing the piezoelectric parameter 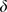 causes the nonlinear capacitance to increase or decrease (dashed curves). The area-specific capacitance has the form 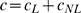, where the motor-independent term, 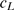, arises from membrane permittivity and historically is termed the “linear capacitance”. The motor-dependent term arises from piezoelectric charge movement and is termed the “non-linear capacitance”. The non-linear capacitance depends on motor stiffness 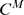 and the mixture fraction 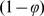. Under conditions of *zero load* 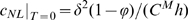 (Fig. 2A), and under *zero-displacement* 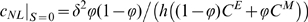 (note, 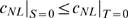). Unlike classic piezoelectrics, the composite admits nonlinear capacitance under *zero-displacement* because active extension of the piezoelectric element is absorbed by contraction of the series elastic element. This is particularly relevant to understanding capacitance measurements in prestin transfected HEK cells where the strain is small [36],[59]. In both cases, the magnitude of 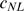 is proportional to 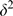 and therefore is also directly related to the isometric force generated by the cell under zero-displacement conditions (Fig. 2B).

#### Frequency-dependent input impedance

The input capacitance of OHCs measured at the base is nearly constant below 1 kHz, but begins to roll-off as the interrogation frequency is increased (Fig. 3 for two ∼55 µm long cells). The roll-off is captured in the model by a loss of space clamp at high frequencies. When cells are deeply hyperpolarized, the voltage dependent component of the capacitance approaches zero, 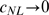, and the model reduces to the cable equation with AC space constant 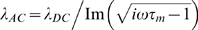 (where 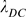 is the standard DC space constant and 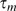 is the passive membrane time constant) [60]. We selected the intracellular resistance to fit the capacitance corner frequency. The resistance value implies that the axial ionic current flows along 21% of the intracellular cross-sectional area (based on electrical conductivity of ∼1.3 S/m). This area is orders of magnitude larger than the annular extra-cisternal space and therefore it is unlikely that all current is channeled strictly along this narrow space as hypothesized previously [61].

#### Zero-load displacement

Fig. 4 compares model predictions to OHC displacement gains in the microchamber [48] and under voltage clamp conditions [15],[62] (cell length range roughly estimated by horizontal error bar). The magnitude of displacement gain reported in Fig. 4 is controlled primarily by the piezoelectric coefficient and the cell mechanical stiffness, while the curved shape is geometrical and arises from the fact that the base of the cell is not electromotile. The microchamber commands the extracellular voltage, *V_p_*, around the basal pole of the cell and, therefore, the intracellular voltage, *V_b_*, is less than present during voltage clamp. This is why the displacements (and gains) in the microchamber configuration (lower curve) are less than in the voltage clamp configuration (upper curve). Voltage clamp data in Fig. 4 was not used to estimate model parameters, yet the simulations correspond well with the experimental observations. Simulations also show the effect of increasing the basal membrane conductance in the microchamber configuration (dashed curve) – a prediction that agrees with previous data collected after application of the efferent transmitter ACh [63] thus further showing the predictive capability of the model. We note that the fast electrical effect of efferent activation occurs even in the absence of additional efferent mediated changes in cell stiffness.

#### Velocity and force vs. frequency

The predictive capability of the model is further illustrated in Fig. 5 comparing velocity predictions for an 80 µm long cell to data collected by Frank et al. in the microchamber (symbols, data for a similar length cell [48]). Model predictions used parameters determined from Figs. 1–4, yet were remarkably similar to these independent experimental observations. OHC velocities increased decade-by-decade over a wide bandwidth, and exhibited a corner frequency, (A, *), above which the velocity flattened and the phase began to roll off. The initial roll-off begins in the model when the piezoelectric force can no longer overpower the viscous drag. The model also predicts a small delay time associated with a dispersive traveling wave along the OHC (∼4 µs for 80 µm cell, not shown). Fig. 5C compares the isometric force predicted by the model for the same cell to experimental data [48]. As expected, the dominant corner frequency observed under isometric force conditions (5C) is higher than observed under zero-load conditions (5A,B) simply because the cell is not moving as much and therefore experiences less electrical and mechanical losses. The same model was used to predict frequency dependent velocity responses for sinusoidal current injection at the apex of the cell (dashed curves) and under physiological hair bundle deflections (dotted curves) leading to adapting apical transduction currents (Eq. 10). It is notable that MET adaptation is predicted to shift the phase of the OHC force and displacement relative to non-adapting current entering the apex of the cell, at least in the mid-frequency band [64]–[66]. Models of the cochlea suggest that a 90° phase shift may be beneficial in the cochlea to align the time of maximum OHCs force generation with that required to increase the vibration of the organ of Corti [67], thus indicating a potential advantage of MET adaptation.

### 2. Electromotility Efficiency

#### Power output vs. velocity

Most experimental data addressing OHC electromotility are collected under conditions of isometric length (Fig. 5C), or zero load (Figs. 2A, 3, 4, 5A–B). In both cases, the mechanical work done by the OHC is zero, and the efficiency is zero. Fig. 6 shows that the peak mechanical efficiency (*) occurs a specific impedance-matched load falling approximately half way between the isometric and zero-load conditions. Specific results shown in Fig. 6 for a 28 µm long OHC at 1 kHz. Although details vary slightly with frequency and cell length, the concept is universal and analogous to the well-known power vs. velocity curves for skeletal muscle cells [30]. Subsequent results (Figs. 7–8) assume that the cochlea efficiently extracts power from OHCs and therefore that the load and the OHC are impedance matched. This implies operation at the peak efficiency, *, in Fig. 6. If true, it is technically feasible, but beyond the present scope, to imply the local impedance within the organ of Corti based on that necessary to match that of the OHCs.

#### Power conversion efficiency vs. frequency

Classical piezoelectricity is thermodynamically conservative [32] and has the potential for 100% efficiency (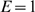). In practice piezoelectric coupling limits efficiency [68], in OHCs to less than 60% [29], due to interplay between the piezoelectric coefficient, stiffness and the electrical permittivity [51],[53],[54],[69]). This loss is shown as “series piezoelectric coupling” at the top of Fig. 7A. Present simulations predict that OHC efficiency is frequency dependent and may reach ∼40% at the best frequency, 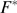. At this optimum frequency, power is lost to fluid viscosity and piezoelectric coupling. We assumed in these simulations that the power delivered by the OHC to fluid viscosity was not of use to the cochlea and therefore causes a reduction in efficiency. It is likely that some of the viscous pumping by OHCs is not lost, but instead may used in cochlea to further amplify vibrations. Therefore, the efficiencies reported here are likely to be a lower bound. In addition to viscous losses, there are two additional intrinsic properties of OHCs that limit efficiency and, in fact, are predicted to be responsible for frequency tuning of the cells. Below 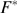, OHC stiffness limits the efficiency. Above 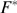, the axial electrical resistance inside the OHC limits the efficiency. Fig. 7B provides efficiency predictions for three different cell lengths, with all other parameters held constant. It is important to note that shorter cells are predicted to be more efficient at high frequencies. This occurs primarily because the space constant of the intracellular electric field shortens with increasing frequency such that 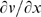 in Eq. 8 becomes nonzero. This couples the electro-mechanical equations and leads to dissipation of electrical power along the length of the cell. Interestingly, simulations for long OHCs exhibited a second peak in efficiency at ultrasonic frequencies (Fig. 7B, near 100 kHz for the 80 µm cell, blue curves), reminiscent of electrical admittances observed previously in isolated OHCs [44].

#### Membrane conductance and efferent control

Activation of the medial olivocochlear efferent bundle reduces mechanical amplification by outer hair cells [4], [70]–[72]. Efferent mediated changes in OHC stiffness [12],[73] likely contribute, but present result also highlight the importance of changes in ionic conductances. Geisler (1974) proposed previously that efferent activation by the brain might alter basal conductance of hair cells and thereby reduce their response [74]. Additional evidence for the conductance proposal comes from the vestibular system and lateral line *in vivo* where activation of the efferent system greatly decreases hair cell receptor gain due to a marked increase in electrical conductance [75],[76], and from the turtle cochlea where efferent activation decreases tuning and receptor gain [77]. These findings are consistent with responses of OHCs to application of the putative efferent transmitter ACh in the dish, where cells increase their displacements evoked in the microchamber configuration [63] – as would be expected with an increase in basolateral membrane conductance noted above (see Fig. 4) due to the additional current that would flow from the microchamber pipette into the cell through the reduced basolateral impedance *Z_b_*.

The present model also addresses how the brain likely controls mechanical power output of OHCs through efferent mediated ionic conductances at the base of the cell. Electrical current entering the MET channels is divided into two parts. The first part drives charge displacement in the lateral wall and is responsible for somatic electromotility through the piezoelectric effect (Eq. 2). The second part of the current exits the base of the cell through conductive ionic channels. If the ion channels are closed (high *Z_b_* case), the part of the MET current driving the somatic motor is maximized. If the ion channels are opened by efferent neurotransmitter (low *Z_b_* case), current will be shunted out the base of the cell and therefore not available to power the motor. This is the reason why opening of basolateral ion channels reduces the efficiency of OHC electrical to mechanical power conversion. This is shown in Fig. 7A as the efficiency drops from the solid curve to the dashed curve. At the same time, the peak efficiency drops (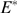 to 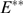) while the best frequency shifts higher (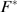 to 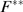). OHCs in the mammalian cochlea do not really experience stimulus frequencies above 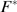 because the traveling wave along the basilar membrane becomes cut off. Hence, the efferent system could reduce power output of OHCs at 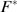 by almost two orders of magnitude simply by shunting the MET power out the basolateral membrane. Solid curves vs. dashed curves in Fig. 7B illustrate that similar effects are present cells of various lengths. Thus, it seems likely that efferent synaptic action upon OHCs sharply attenuates the mechanical power output at best frequency by shunting the electrical power input via the MET current to ground.

#### OHC length vs. best frequency

Shorter cells exhibited their best efficiency at high frequencies while longer cells exhibited their best efficiency at low frequencies. Fig. 8A shows OHC length vs. maximum efficiency frequency (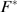) along with data correlating the lengths of OHCs in the cochlea to the place principle describing the best frequency of sound sensation. Above 1 kHz, the morphological relationship between OHC length and physiological best frequency in the cochlea [78] is bracketed by the best efficiency predicted here. These results are consistent with the hypothesis that OHC lengths are matched to the frequency requirements at their location in the cochlea. Interestingly, if we consider the peak efficiencies over all hair cells studied, the analysis predicts that hair cells tuned to ∼3–4 kHz are the most efficient (Fig. 8B). This might augment the high efficiency of the middle ear in this frequency band [79],[80] and further accentuate sensitivity to damage by acoustic overexposure.

## Discussion

There are four major observations that can be drawn from the present work. The first addresses how OHCs operate at high frequencies given their electrical capacitance [9], [62], [81]–[83]. Capacitance is thermodynamically conservative and present results confirm that the ability of OHCs to supply mechanical power to the cochlea is not limited by electrical capacitance [84], even at frequencies much higher than the membrane time constant (e.g. Fig. 7). This is true because capacitance is not dissipative. Instead, present results suggest the most serious factor that may limit power output by OHCs is how well the “impedance” of the hair cell is matched to that of the cochlear partition (e.g. Fig. 6). OHCs driving against an excessively stiff cochlear partition, for example, would be inefficient.

The second observation is that OHCs may be tuned to maximize their power output at a best frequency, albeit broadly tuned. Although OHC displacement and force are quite flat over a broad range of frequencies when driven by voltage (e.g. Fig. 5, present model and published data [48]), OHC power output is tuned when one considers the mechanical power output relative to the electrical power input. The predicted tuning is dependent upon cell length and correlates with the cochlear place principle [78], thus indicating that tuning of OHCs may contribute to the sharp mechanical and afferent neural tuning in the living cochlea.

The third observation addresses how the MET channels would be expected to further tune output of the somatic motor. MET adaptation generates high-pass filtered MET currents [47],[66],[85],[86]. Since the filtering is upstream of the somatic motor it would further sharpen tuning of OHC somatic motor output by attenuating low-frequency amplification. In the context of the organ of Corti, MET adaptation would also be expected to alter the phase of the OHC force possibly to maximize power input to the cochlea near the best frequency [67] and, additionally, might introduce a non-optimal phase that would sharply attenuate cochlear gain at both low and high frequencies. Because of these factors, the influence of tuning in isolated OHCs on tuning curves in the cochlea would be expected to be even more significant than implied by the OHC motor efficiency alone (Fig. 7).

The fourth observation is that OHC somatic power output may be controlled by the brain *via* efferent activated ionic conductance(s). The model predicts that increasing the conductance of the basal pole would reduce OHC power output and tuning, thus providing a plausible explanation for a fast mechanism that may be used by the brain to control both sensitivity and frequency selectivity of hearing (e.g. Fig. 7).

Finally, it is important to note that the OHC somatic motor is not present in non-mammals, yet these animals also exhibit many of the properties of the mammalian cochlear amplifier [87],[88]. The MET apparatus itself is clearly a key contributor to hair bundle motility and amplification [9],[89]. In addition, there is an MET-independent component of hair bundle motility driven by voltage [10]. This voltage-dependent component has analogy to the somatic motility addressed here, and may be involved in tuning and the power stroke of hair bundle motility with potential relevance to active bundle amplification in high frequency hearing organs [90]. These hair-bundle features occur upstream of the somatic motor and the two clearly interact with each other *via* micromechanical environment and electrical fields [91].
